# Cocaine-Induced Lung Damage and Uncommon Involvement of the Basal Ganglia

**DOI:** 10.7759/cureus.53330

**Published:** 2024-01-31

**Authors:** Hamid Ziani, Siham Nasri, Imane Kamaoui, Imane Skiker

**Affiliations:** 1 Radiology, Mohammed VI University Hospital, Faculty of Medicine, University Mohammed First, Oujda, MAR

**Keywords:** mri, ct, lung, basal ganglia, cocaine-induced intoxication

## Abstract

Cocaine use is responsible for multiorgan damage, including the brain and lungs. Bilateral and symmetrical involvement of the basal ganglia may be due to toxic, metabolic, vascular, inflammatory, infectious, or tumoral causes. Cocaine-related encephalopathy mainly affects the white matter, while basal ganglia involvement is an uncommon finding. Cocaine-induced lung damage varies clinically and even radiologically, with signs that lack specificity. The diagnosis of cocaine-induced lung or brain injury is based on suggestive radiological signs in the context of cocaine consumption and after the elimination of other etiologies likely to present the same patterns. The context of cocaine use is often not spontaneously declared, making diagnosis more complicated. We report the case of a 28-year-old male patient, with a history of freebase cocaine use, admitted to the emergency room in severe coma with respiratory distress. Brain MRI showed bilateral and symmetrical abnormalities of the basal ganglia. A chest CT scan revealed interstitial lung damage dominated by the ground-glass pattern. The urine toxicology test was positive for cocaine. Cocaine-related lesions can be reversible, and therapeutic management is essentially based on supportive care.

## Introduction

Toxic lung and brain injury following illicit drug use is well known in the literature and current medical practice. Several drugs have been incriminated, including cocaine. Cocaine is considered the second most widely used drug in the world, particularly among young men, with an average age of 32 years [1]. Cocaine-induced lung or brain damage can be acute, leading to admission to the emergency department in a state of respiratory distress and/or coma. The pathophysiological mechanisms behind cocaine-related damage vary, including direct effects of oxidative stress, vascular abnormalities, adrenergic overstimulation, and metabolic changes. Thus, even the clinical consequences are variable, as are the radiological findings [2]. Through this case report and literature review, we illustrate the cocaine-induced bilateral involvement of the basal ganglia, which is uncommon, and show the CT signs of lung damage in the same context.

## Case presentation

A 28-year-old male patient, with no particular medical history, a chronic smoker, with a history of freebase cocaine use, was admitted to the emergency room in a severe coma with respiratory distress. Clinical examination revealed a Glasgow Coma Scale score of 3, bilateral mydriasis, a blood pressure of 105/66 mmHg, a heart rate of 78 beats/minute, and peripheral capillary oxygen saturation (SpO_2_) of 63%. The patient was apyretic and had cyanosis of the fingers. His blood glucose level was normal.

Resuscitation measures were initiated immediately, including intubation and assist-control ventilation as well as hemodynamic and respiratory monitoring. Laboratory tests did not show any metabolic abnormalities. A brain CT scan initially showed no abnormality. A chest X-ray taken at the patient’s bed revealed acute interstitial lung disease.

**Figure 1:**
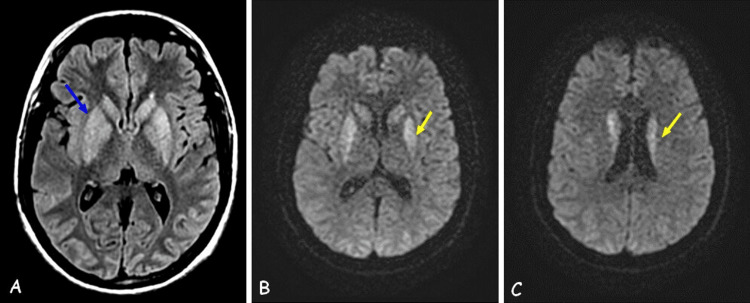
Brain MRI showing bilateral and symmetrical T2 and FLAIR (A) hyperintensity of the basal ganglia with diffusion restriction (B and C). Blue arrow: T2 FLAIR hyperintensity of the basal ganglia. Yellow arrows: DWI hyperintensity of the basal ganglia. DWI: diffusion-weighted imaging; FLAIR: fluid-attenuated inversion recovery

Brain MRI showed bilateral and symmetrical T2 and fluid-attenuated inversion recovery hyperintensity of the basal ganglia with diffusion restriction, without any abnormal enhancement (Figure 1).

**Figure 2:**
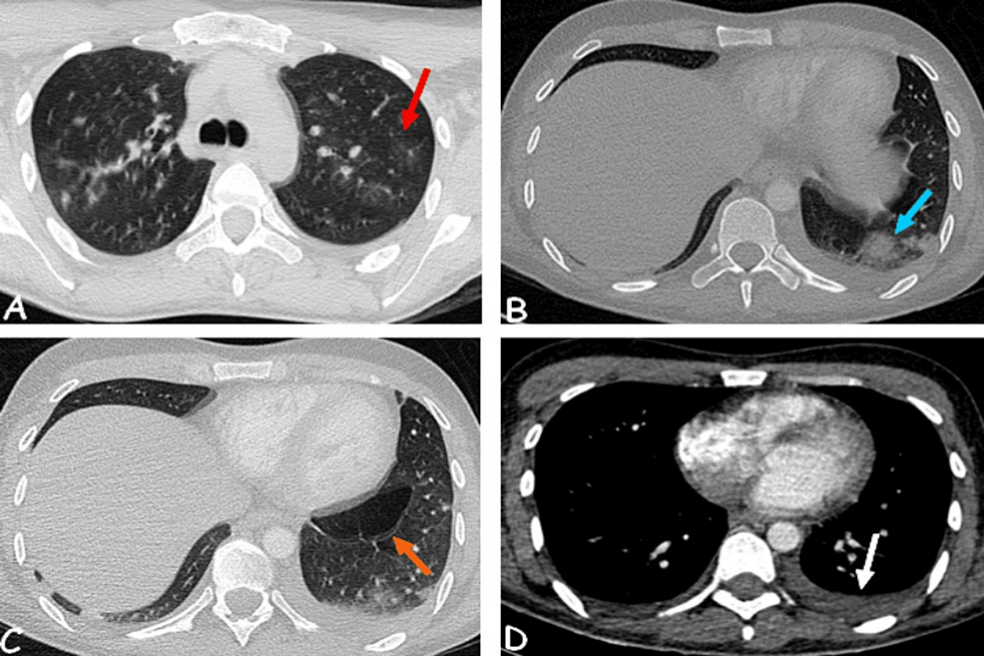
Chest CT scan showing bilateral ground-glass opacities (A), consolidations (B), emphysematous bullae (C), and pleural effusion (D). Red arrow: ground-glass pattern. Blue arrow: pulmonary consolidation. Orange arrow: emphysematous bullae. White arrow: left pleural effusion.

To better characterize the lung involvement, a chest CT scan was performed, which showed bilateral ground-glass opacities with a more pronounced distribution in the upper lobes, peripheral bilateral consolidations, emphysematous bullae in the left lower lobe, and bilateral pleural effusion (Figure 2).

The patient tested negative for COVID-19. The urine toxicology test was positive for cocaine. It was negative for morphine and amphetamine. The patient received supportive care, including oxygen therapy, optimization of fluid and electrolyte status, thromboembolic prevention, and stress ulcer prophylaxis. No antidote was administered. He was extubated and regained consciousness after five days of prompt resuscitation. The patient was subsequently referred to a psychiatric and addictology consultation for dependence management.

## Discussion

Cocaine is considered the most common cause of drug-related deaths, and crack is the most widely used form [1]. Cocaine abuse is responsible for multiorgan damage involving the heart, brain, lungs, kidneys, liver, and other organs depending on the route of administration [2].

Pathophysiological mechanisms described to explain cocaine-induced lung injury are direct toxic effect and thermal damage to the airways, alteration of the alveolar-capillary membrane (increased pulmonary capillary permeability leading to lesional pulmonary edema), inflammation and immunomodulation (initiated by a decrease in interferon-gamma (IFN-γ) and interleukin-8 production by peripheral blood lymphocytes, given that IFN-γ is involved in the pathogenesis of interstitial lung disease), vasoconstriction (anoxic damage to lung endothelial or epithelial cells), and barotrauma [1,3]. Cocaine-induced pulmonary disorders reported in the literature include acute pulmonary edema, alveolar hemorrhage, pneumothorax, organizing and eosinophilic pneumonia, pulmonary infarction, and “crack lung” [4]. Crack lung is an acute pulmonary syndrome that occurs following the inhalation of freebase cocaine. Its clinical signs are variable, including respiratory distress, hemoptysis, and chest pain with or without fever [5]. Other signs may be associated, notably those due to the adrenergic effect of cocaine, such as hypertension, tachycardia, and mydriasis [6].

There is no specific biological parameter for cocaine-induced injury and plasma hypereosinophilia is inconstant. Urinary benzoylecgonine is positive for 24 to 48 hours after acute cocaine use and remains positive for up to a few weeks in the case of chronic use [7]. The diagnosis of cocaine-induced lung and brain damage is based on suggestive radiological signs in the context of cocaine consumption and after the elimination of other etiologies likely to present the same patterns. The context of cocaine use is often not spontaneously declared, making diagnosis more complicated. Some physical signs have been described that may point to cocaine use, including burned fingertips and black sputum [1].

The CT scan shows a ground-glass appearance, consolidations, regular thickening of the septal lines with a crazy paving appearance, paraseptal emphysema, and centrilobular nodules. These abnormalities affect the upper, middle, and lower parts of the lung as well as the parahilar region. A fluid pleural effusion may be present. These abnormalities would indicate alveolar hemorrhage, acute hypersensitivity pneumonitis, acute eosinophilic pneumonitis, or acute respiratory distress syndrome. The term crack lung has been used, given the difficulty of differentiating between these entities, which may have the same radiological patterns [1,8].

The bilateral and symmetrical involvement of the basal ganglia described in several types of intoxication is not a common sign of cocaine-induced brain damage. On MRI, white matter abnormalities are the most frequently described signs of cocaine-induced encephalopathy. The involvement of the basal ganglia may be explained by vascular mechanisms that have yet to be studied [9,10].

Management of cocaine-induced lung and brain injury is essentially based on oxygen therapy and supportive care. The use of corticosteroids has been described in some cases. Clinical improvement can be observed within the first 72 hours [3,4].

## Conclusions

Given the fact that the context of cocaine use is often not spontaneously declared, diagnosis of cocaine-induced lung and brain damage can be difficult as the clinical and radiological signs are not specific. As illustrated in this case, cocaine can also be responsible for bilateral and symmetrical lesions of the basal ganglia, which have not been widely described in the literature.
